# Unlocking the Secrets: Exploring the Biochemical Correlates of Suicidal Thoughts and Behaviors in Adults with Autism Spectrum Conditions

**DOI:** 10.3390/biomedicines11061600

**Published:** 2023-05-31

**Authors:** Ivan Mirko Cremone, Benedetta Nardi, Giulia Amatori, Lionella Palego, Dario Baroni, Danila Casagrande, Enrico Massimetti, Laura Betti, Gino Giannaccini, Liliana Dell'Osso, Barbara Carpita

**Affiliations:** 1 Department of Clinical and Experimental Medicine, University of Pisa, via Roma 67, 56126 Pisa, Italy; ivan.cremone@gmail.com (I.M.C.); benedetta.nardi@live.it (B.N.); giulia.amatori@libero.it (G.A.); danilacasagrande@yahoo.it (D.C.); liliana.dellosso@unipi.it (L.D.); 2 Department of Pharmacy, University of Pisa, 56126 Pisa, Italy; lionella.palego@unipi.it (L.P.); dariobaroni@ymail.com (D.B.); laura.betti@unipi.it (L.B.); gino.giannaccini@unipi.it (G.G.); 3 ASST Bergamo Ovest, SSD Psychiatric Diagnosis and Treatment Service, 24047 Treviglio, Italy; e.massimetti@yahoo.it

**Keywords:** autism spectrum disorder, brain derived neurotrophic factor, biomarkers, homocysteine, interleukin-6, inflammation, kynurenine pathway, serotonin, suicidality, suicidal behavior, tryptophan

## Abstract

Involving 1 million people a year, suicide represents one of the major topics of psychiatric research. Despite the focus in recent years on neurobiological underpinnings, understanding and predicting suicide remains a challenge. Many sociodemographical risk factors and prognostic markers have been proposed but they have poor predictive accuracy. Biomarkers can provide essential information acting as predictive indicators, providing proof of treatment response and proposing potential targets while offering more assurance than psychological measures. In this framework, the aim of this study is to open the way in this field and evaluate the correlation between blood levels of serotonin, brain derived neurotrophic factor, tryptophan and its metabolites, IL-6 and homocysteine levels and suicidality. Blood samples were taken from 24 adults with autism, their first-degree relatives, and 24 controls. Biochemical parameters were measured with enzyme-linked immunosorbent assays. Suicidality was measured through selected items of the MOODS-SR. Here we confirm the link between suicidality and autism and provide more evidence regarding the association of suicidality with increased homocysteine (0.278) and IL-6 (0.487) levels and decreased tryptophan (−0.132) and kynurenic acid (−0.253) ones. Our results suggest a possible transnosographic association between these biochemical parameters and increased suicide risk.

## 1. Introduction

According to the World Health Organization, approximately 1 million individuals worldwide commit suicide each year [1]. Suicide represents one of the major topics of psychiatric research; however, its definition varies in the literature, and there is still an open debate over standardizing its nomenclature, particularly for elements regarding the intentionality [2,3]. A commonly used definition for suicide is a “fatal self-inflicted self-destructive act with explicit or inferred intent to die” [4], but that only represents a phenomenon that lies at the extreme end of a continuum of actions that are collectively referred to as suicidal behaviors, which also includes suicidal ideation and attempted suicide [3,5]. In particular, suicidal ideation is described as contemplating or planning to commit suicide, while suicide attempts are non-lethal, self-directed injurious behavior with an intent to die [6]. Despite there being a great amount of studies in recent years focusing on the possible neurobiological underpinnings of suicidal ideation and behaviors, to date, understanding and predicting suicidal attempts remains a challenge. Although a significant number of clinical, psychosocial, sociodemographical risk factors and prognostic markers have been proposed [7], they typically have poor predictive accuracy [8] and are frequently unmodifiable [9]. Moreover, the prediction of suicidal attempts relies on subjective indicators, such as patient accounts of thoughts, behavior, and family history [10,11,12]. For all these reasons, the literature stresses the need for more objective indicators in order to facilitate the development of effective techniques for prevention and intervention, as well as the improvement in the ability of prediction, risk screening, and evaluation of suicidality [9]. Furthermore, a better understanding of the neurobiological processes of suicidal thoughts and behaviors are also needed to provide unambiguous knowledge of the pathophysiology of suicide and find new treatment targets and strategies [13].

In this context, biomarkers can offer crucial information by serving as predictive indicators, providing proof of treatment response and proposing potential targets for new treatment approaches [6]. Biomarkers, which are broadly referred to as objective indicators of a biological state or condition, may be quantified in a variety of ways, such as changes in protein expression, epigenetic markers, and metabolomic alterations that can be found in both the central nervous system (CNS) and peripheral nervous system (PNS) [14]. In a clinical setting, biomarkers for suicidality (both ideation and attempts) may offer more assurance regarding the diagnosis or the course of therapy rather than psychological measures alone [6].

The recent literature has highlighted the role of neuroinflammation as a key player in the pathophysiology of suicidality, including both suicidal ideation and behaviors [15,16,17,18,19]; this evidence is in line with items of research that have consistently observed central and peripheral chronic inflammation in many psychiatric disorders [20]. In fact, regardless of their age or gender, individuals who manifested suicidal thoughts and attempts are reported to have higher levels of inflammation and oxidative stress markers in both the central nervous system (CNS) and peripheral tissues [21,22,23]. In addition, the fact that the stress response is significantly correlated with the immune system strongly supports the theory that investigating inflammation could be a promising path to grasp a better understanding of suicidality [13,24,25]. However, to date, results concerning the role that different mediators of inflammation play in the etiopathology of suicide are still mixed [26,27]. Numerous items of research have emphasized the link between suicidality and inflammatory states, highlighting the presence of higher levels of proinflammatory cytokines in the blood, the cerebrospinal fluid (CSF), and post-mortem brain [13,28,29,30,31,32,33,34,35] of suicide attempters, indicating that cytokine activation may affect vulnerable people’s tendency to suicide. In particular, among the cytokines, the most proposed potential markers of suicidality are interleukin-1 beta (IL-1β) and interleukin-6 (IL-6), which have been repeatedly shown to be elevated in the blood and the CSF of suicidal patients [17,21,36,37,38,39,40]. IL-6 is a member of the proinflammatory cytokine family that not only stimulates the production of several proteins involved in acute inflammation processes, but also plays a significant role in human cell differentiation and proliferation [41]. Moreover, IL-6 has been linked to the physiological homeostasis of neural tissue and to the pathogenesis of many diseases with significant neuropathological changes [42], while other studies have suggested that increased amounts of IL-6 may be able to modify the behavioral output from the brain and may influence neuronal plasticity, neurogenesis, and neurotransmission, even though the precise mechanism has not yet been clarified [43,44,45]. Additionally, IL-6 has been linked to mental illnesses such as anxiety, despair, and suicide ideation [46], and numerous studies have historically reported a correlation between IL-6 levels and suicidality both in adults and adolescents [47,48,49].

Numerous items of research have emphasized how the stimulation of the kynurenine (KYN) pathway of tryptophan (TRP) degradation, caused by neuroinflammation, may lead to an imbalance of the pathway metabolites, some of which may directly influence the manifestation of suicidal symptoms via the regulation of glutamate neurotransmission [50]. Moreover, the stimulation of the kynurenine (KYN) pathway leads to a reduction of TRP that ultimately causes a depletion of serotonin (5-HT) and melatonin [51], which is not only associated with an increase in aggressive and impulsive behaviors [52] but is also reported to be one of the major findings that differentiated biomarkers of suicide from those of other psychiatric disorders [53,54]. Additionally, kynurenic acid (KYNA)’s enhanced metabolism into the neurotoxic NMDA receptor agonist, quinolinic acid (QUIN), may lead to an overactivation of the glutamatergic system, which in turn affect brain-derived neurotrophic factor (BDNF) synthesis, with an eventual impact on neuroplasticity and cognition [55]. Regardless of the presence of co-occurring mood disorders, higher levels of QUIN were found in the cerebrospinal fluid (CSF) of patients who attempted suicide and those who had suicidal thoughts [51,56] and were also correlated to a higher score on the Suicide Intent Scale [51,57].

On the other hand, as reported by multiple twin and family studies, genetics may be one of the variables influencing the risk of suicide [58]. In particular, the genetic component for suicide also appears to be shared with the inheritance of psychiatric disorders [59,60], and a recent genome-wide association study revealed an intriguing correlation between suicide attempts and depressive symptoms, neuroticism, schizophrenia, insomnia, and major depressive disorder [61,62]. However, it is still difficult to pinpoint the genetic basis for suicide behaviors. To date, a variety of single nucleotide polymorphisms have been identified as being associated with suicide in a genome-wide association study with polygenic risk scores [63,64,65], but the heritability estimated from these common variants only ranges between 4% and 4.6% [62,66], thus suggesting a complex biological background and a potential multifactorial origin behind these behaviors.

On the other hand, results from clinical and psychopathologic studies have stressed how one of the neuropsychiatric conditions most frequently associated with increased suicidal risk is the autism spectrum disorder (ASD) or also subthreshold autistic traits (AT) [67,68,69,70]. ASD is a neurodevelopmental disorder characterized by severe and pervasive impairment in reciprocal socialization, qualitative impairment in communication, and repetitive or odd behavior. Recently, it has been determined that autism is a disorder of genetic [71] and neurological origin with defects in the coordinated functioning of various brain regions [72,73]. Many studies have reported a lower synchronization of the activation (or functional connectivity) between frontal and posterior brain regions in ASD subjects in a wide variety of tasks [74,75,76,77,78,79,80,81], including language comprehension tasks [82]. Moreover, an aberrant trajectory of brain development is often reported, which is consistent with autism being a developmental condition. Many researchers have reported cortico–cortical connection abnormalities in ASD subjects, possibly caused by abnormalities in any of numerous brain development pathways [76,82,83,84,85,86,87,88], and defection in the pruning of synapses during later stages of neural development [89,90]. These abnormalities in maturational processes are in line the observation, during the early stages of development, of larger brain sizes in autistic patients [91,92], especially in the frontal cortex [93], and loss of cerebellar Purkinje cells, which is correlated with the severity of social impairments [94]. Additionally, many authors have highlighted increased brain size and white matter volumetric abnormalities [95], particularly in the frontal lobes, in young children with autism [92,93], impaired white matter connectivity, that can be assumed to be a neural substrate for socio-emotional dysfunction in ASD, specifically for social signal interpretation in social interaction [96,97,98], and reduction in the amount of white matter over adolescence and adulthood [83,99,100]. Subthreshold autistic traits have been firstly identified among first degree relatives of ASD probands, where they are known under the name of Broad autism phenotype (BAP), a label that stresses the possible common genetic underpinnings between different presentations of the autism spectrum [101]. However, recent literature has highlighted how AT seem to be continuously distributed from the clinical to general population, being particularly frequent among psychiatric patients with other kinds of psychiatric disorders [65,102,103,104,105,106,107,108,109], leading to the formulation of a neurodevelopemental hypothesis for psychiatric disorders [110]. Indeed, a growing body of research is suggesting that not only ASD subjects share an increased risk of suicidal thoughts and behaviors [95,111,112,113,114,115,116,117,118], but also that the presence of elevated AT in non-autistic populations represent a confirmed risk factor for lifetime suicidality [66,119,120,121]. However, to the best of our knowledge, no study has specifically investigated the biochemical correlates of suicidal ideation and behaviors among adult subjects in the autism spectrum.

In this framework, the aim of this study was to evaluate how the blood levels of 5-HT, BDNF, TRP, and its metabolites of the KYN pathway (specifically, KYN, KYNA, and QUIN), as well as the levels of IL-6, and of homocysteine (HCY) (as a potential marker of altered trans-sulfuration and transmethylation pathways, eventually leading to impaired redox balance and DNA methylation) may be associated with suicidality in a sample of adult ASD patients, their first-degree relatives (BAP), and healthy controls (HC).

## 2. Materials and Methods

For the study, a sample of 24 adults with autism, their first-degree relatives, and 24 controls was recruited. A blood sample was taken from each subject and all biochemical parameters were measured with enzyme-linked immunosorbent assays. Suicidality was measured through selected items of the MOODS-SR questionnaire.

### 2.1. Recruitment Procedures

ASD subjects (ASD group) were recruited among out-patient and in-patients followed at the Psychiatric Department of the Azienda Ospedaliera Universitaria Pisana (AOUP), University of Pisa. In order to participate in the study, the patient group must have received an ASD diagnosis, be aged at least 18 and not be aged below 65 years, and have no or only milder intellectual impairment and/or language developmental alteration. During the recruitment procedures, for each patient the participation of a parent or a sibling was requested: the relatives’ group was labeled as the “BAP group”. Being unable to complete the evaluations due to a linguistic or intellectual disability, having a diagnosis of schizophrenia, a substance use disorder, a neurodegenerative disease, or any other relevant medical or neurological disorder were the main exclusion criteria for both groups. Additionally, BAP subjects were excluded if they had been given a DSM-5 diagnosis of ASD or another neurodevelopmental disorder and if they were younger than 18 years old. The HC group was recruited on a voluntary basis. Exclusion criteria for this group were the same used for the ASD group, with the exception that subjects in the CTL groups were also excluded if they received a diagnosis of a psychiatric disorder according to DSM-5 criteria.

Psychometric measures and a structured clinical interview were used to evaluate each individual. A blood sample was taken from each participant in order to conduct the biochemical assessment. All subjects received clear information about the study and had the opportunity to ask questions before providing a written informed consent. The present research was led in accordance with the declaration of Helsinki, and all procedures were approved by the local ethical committee.

### 2.2. Psychometric Instruments

All subjects were clinically evaluated by trained psychiatrists, and the ASD diagnosis was confirmed according to DSM-5 criteria. The Structured Clinical Interview for DSM-5 disorders (SCID-5) was employed for evaluating the eventual presence of other comorbid psychiatric conditions. Mood symptoms and suicidality scores were evaluated by means of the Mood Spectrum Questionnaire (MOODS-SR).

#### 2.2.1. The Structured Clinical Interview for DSM-5 Disorders (SCID-5)

The SCID-5 is the gold standard structured clinical interview for investigating the presence of major psychiatric disorders according to DSM-5 [122]. It must be administered by trained mental health professionals. It is composed of 10 independent modules; the sequence of questions follows the order of the related diagnostic manual (DSM-5), and the different items of each module guide the interviewer through the evaluation of the presence of symptoms that may satisfy the diagnostic criteria.

#### 2.2.2. The Mood Spectrum Questionnaire (MOODS-SR)

The MOODS-SR is a questionnaire designed to assess a wide range of temperamental characteristics and mood symptoms throughout the course of a lifetime. It has 160 items and is divided into Manic Component, Depressive Component, and Rhithmicity domains. There are two possible answers for each item: “Yes” and “No.” The instrument’s Cronbach’s alphas for the Italian translation ranged from 0.72 to 0.92. The MOODS-SR was used in earlier studies to assess suicidality (with subsections for suicidal ideation and behaviors) [67,70,123], through the instrument’s items 102 to 107.

### 2.3. Biochemical Evaluations

Biochemical analyses were conducted on the blood samples of all participants, which were collected in the mornings after at least 12 h of fasting in K3EDTA vacutainer tubes or in clot activator tubes for serum separation. Platelet rich plasma (PRP) was separated from other blood elements by centrifuging the samples at 150× *g* for 15 min, at room temperature (RT). Subsequently, platelet poor plasma (PPP) and platelet pellets were obtained by transferring the PRP aliquots in falcon tubes and centrifuging them again at 1500× *g* for 15 min, at RT. In order to obtain serum aliquots, the samples in clot activator tubes were centrifuged only one time at 1500× *g* for 15 min, at RT. Finally, the obtained PRP and serum aliquots were transferred to high-quality, low-binding protein Eppendorf Safe-Lock test tubes, while platelet pellets remained in Falcon tubes. All these samples were maintained at −80 °C until the day of the assay. All biochemical parameters were measured with enzyme-linked immunosorbent assays (ELISA). IL-6 and BDNF levels were measured in the PPP with sandwich ELISA kits (Picokine IL-6 assay, Boster Biological Technology, Pleasanton, CA, USA and Biosensis, mature BDNF RapidTM, Thebarton, Australia) featuring a 96-well microplate. All measures were performed in duplicate. The ELISA kits featured a primary monoclonal anti-IL-6 or anti-BDNF antibody, a second biotinylated antibody, and a streptavidin-biotin complex. Levels of HCY, 5-HT, TRP, and KYN pathway metabolites (KYN, QUIN, KYNA) were measured through indirect competitive ELISA kits (ImmuSmol, Bordeaux, France). All these parameters were measured in PPP, with the exception of KYNA, which, according to the kit instruction, was measured in serum. For 5-HT, intra-platelet levels were also measured. These kinds of ELISA kits featured microplates pre-coated with quotes of the same analyte object of investigation; in order to perform the competitive essay, the analyte as well as a first specific antibody were added to each well. Subsequently, for the detection reaction, a second a second biotinylated antibody linked to horseradish peroxidase (HRP) was added, followed by the HRP substrate, 3,3′,5,5′-tetramethylbenzidine (TMB). Regarding HCY, due to the risk of matrix effects if measured directly, according to the kit instruction PPP aliquots were incubated with an enzyme reaction mixture containing the S-adenosyl-L-homocysteine hydrolase enzyme and its substrate adenosine/dithiothreitol (DTT) before the assay, thus transforming HCY to S-adenosyl-homocysteine. Regarding the measurement of 5-HT intra-platelet levels (ng/mL), in order to avoid eventual biases associated with individual differences in platelet count, we proceeded according to Bradford’s method [124], normalizing the obtained concentrations for total proteins (mg/mL). The normalized concentrations were reported as ng/mg protein.

### 2.4. Statistical Analysis

**Figure 1:**
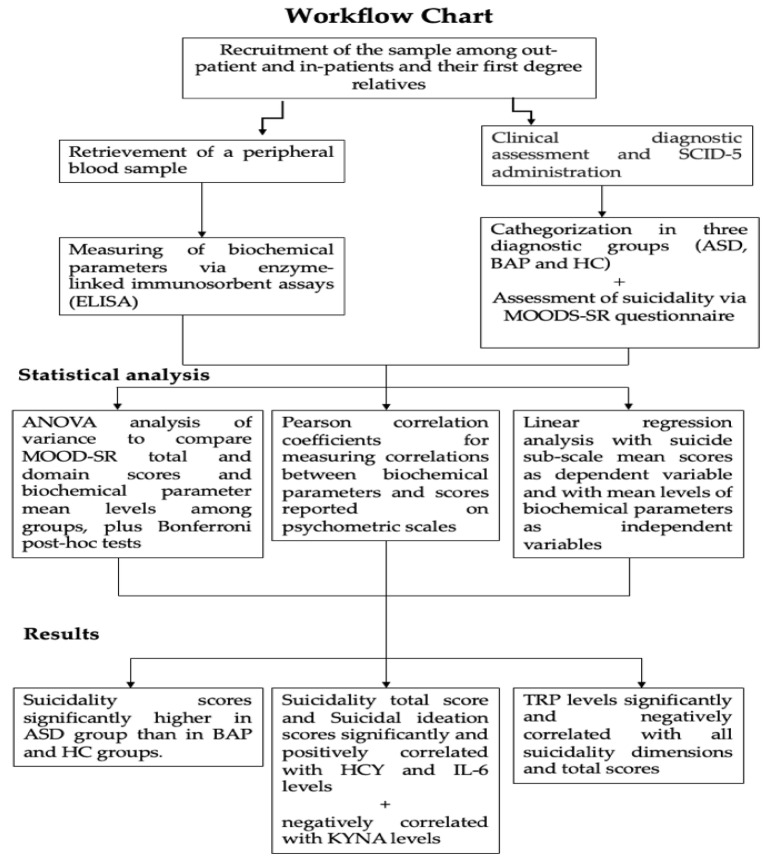
Summarizing flowchart.

We performed an ANOVA analysis of variance in order to compare MOOD-SR total and domain scores, as well as biochemical parameter mean levels among groups, followed by Bonferroni post-hoc tests. Pearson correlation coefficients were calculated for measuring the correlations between biochemical parameters and scores reported on psychometric scales. In order to evaluate eventual biochemical parameters statistically predictive of suicidality in the sample, a linear regression analysis was performed with the suicide sub-scale mean scores as the dependent variable and with mean levels of biochemical parameters as independent variables. All the analyses were performed using SPSS, version 24 (IBM Corp., Armonk, NY, USA, 2016). For biochemical assays, calibration curves and the regression analyses were performed by means of GraphPad Prism (Version 7.0, San Diego, CA, USA). See Figure 1. 

## 3. Results

Comparison of socio-demographic variables, as well as other clinical and psychopathological features of the sample, are described elsewhere [125,126,127]. Considering MOODS-SR scores, the ANOVA analysis showed significant differences among groups on MOODS Total (F = 33.62; dF = 2; *p* < 0.001) as well as on MOODS manic component (F = 18.13; dF = 2; *p* < 0.001), depressive component (F = 35.39; dF = 2; *p* < 0.001), and Rhythmicity (F = 12.74; dF = 2; *p* < 0.001). According to Bonferroni post-hoc tests, the ASD group reported higher MOODS-SR totals and domain scores than the BAP group, which in turn reported significantly higher scores than the HC group (see Table 1).

Suicidality scores were also significantly different among groups considering total score (F = 14.81; dF = 2; *p* < 0.001), suicidal ideation score (F = 12.22; dF = 2; *p* < 0.001), and suicidal behavior score (F = 8.62; dF = 2; *p* < 0.001). In particular, according to Bonferroni post-hoc tests, all suicidality scores were significantly higher in the ASD group than in the BAP and HC groups, without significant differences between the latter two (see Table 2).

Results from Pearson correlation coefficient analysis between biochemical parameters and MOODS-SR scores showed significant positive correlations of IL-6 levels and MOODS total and domain scores, with the exception of the MOODS-SR manic component. Significant positive correlations were also reported between HCY levels and MOODS-SR depressive component scores. Moreover, significant negative correlations were reported between all MOODS-SR domain scores and both KYNA and TRP levels (see Table 3).

**Figure 2:**
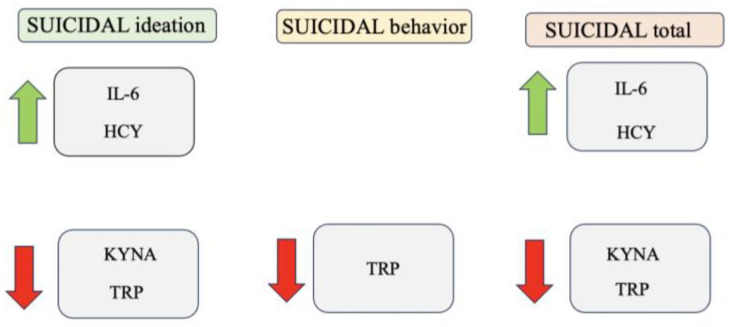
Schematic representation of associations between suicidality and biochemical variables.

Regarding correlations with suicidality scores, we found that suicidality total score and suicidal ideation scores were significantly and positively correlated with both HCY and IL-6 levels, while they were negatively correlated with KYNA concentrations. Finally, TRP levels were significantly and negatively correlated with all suicidality dimensions and total scores (see Table 4 and Figure 2).

According to the linear regression analysis, HCY levels were identified among all the biochemical parameters as significant positive predictors of higher suicidality score (see Table 5).

Our results are in line with the previous literature suggesting an involvement of inflammation in the development and maintenance of various psychiatric disorders, in this case suicidal behaviors.

## 4. Discussion

Our results highlighted higher suicidality scores in the ASD group compared to the BAP and HC groups, while no differences were reported between the latter. Concerning the biochemical parameters, a significantly negative correlation between KYNA, TRP, and all MOODS-SR domain scores, as well as a significant negative correlation between the same parameters and suicidality scores, was highlighted. Contrarywise, we found a positive correlation between the proinflammatory cytokine IL-6 and MOODS-SR total and domain scores, as well as a significant positive correlation between IL-6 and suicidality and a positive correlation between HCY and the MOODS-SR depressive component, as well as with suicidality scores.

This evidence is in line with previous items of research that have reported an increased suicidality risk in ASD populations [58,95,111,112,113,114,115,116,117,118,119,120,121]. The association between ASD and suicidality has long been discussed, and a number of factors contributing to the correlation have been identified. According to the “interpersonal theory of suicide”, the social difficulties and their consequences (such as loneliness [128,129], peer victimization [130,131], adoption of camouflaging behaviors [132,133,134]), and the resulting lack of meaningful social connections, may increase the risk of contemplating suicide. A recent study that investigated the relationship between AT and suicidality highlighted how the deficits in non-verbal communication and the restricted interest and ruminations were significantly and positively correlated with suicidality scores [67]. However, our findings, reporting similar levels of suicidality between BAP and HC groups, partially differ from those of another study that also highlighted increased suicidality scores mong subjects with subthreshold AT. This difference may be explained by the more limited sample size of the current study, and/or by the different methods of selection of the subthreshold AT traits/BAP group [70].

**Figure 3:**
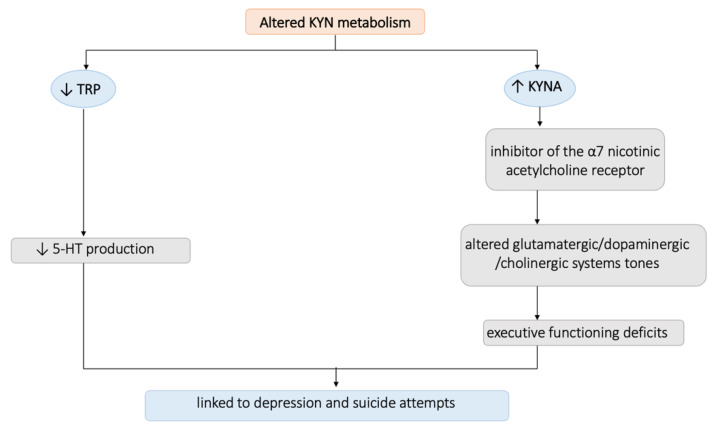
Summarizing figure on the altered KYN metabolism’s contribution to suicidality.

Results from the evaluation of the biochemical parameters highlighted a significantly negative correlation between KYNA, TRP, and all MOODS-SR domain scores, as well as a significant negative correlation between the same parameters and suicidality scores. KYNA is a metabolite of the TRP metabolic system, is robustly synthesized in the endothelium, and its serum levels correlate with homocysteine [135]. These evidences are in line with the strong and widespread correlation between the KYN pathway and depression that has been reported in the scientific literature, starting from its first appearance in The Lancet in 1969 with the name of the “serotonin hypothesis” [136], which claimed that the increment of TRP-KYN pathway metabolism, caused by the activation of the hepatic TRP-pyrrolase (TDO) due to elevated steroid levels, had a great impact on various neurological functions [137,138]. These statements have been confirmed and widened by the growing number of studies that are reporting an alteration of the TRP-KYN metabolism in a wide range of illnesses, including neoplastic, immunologic, neurological, and psychiatric disorders [18,139,140,141]. Subsequently, various studies have evaluated the levels of the KYN pathways in patients suffering from mood disorders. In particular, a recent meta-analysis including 22 studies reported that patients suffering from depression showed reduced levels of KYNA and KYN and increased levels of QUIN [142]. The first evidence of a link between the dysregulation of the KYN pathway and suicidality emerged many years later, in a study that highlighted elevated plasma KYN levels in depressed patients who had attempted suicide compared to patient who never attempted [143]. However, in agreement with our findings, opposite results came from more recent studies. In particular, recent research reported a 40% decrease in plasma TRP levels in suicidal adolescents with major depression (MDD), compared to non-suicidal individuals with MDD and HC [144], which is in agreement with many other studies that have linked lower peripheral TRP levels to suicide tendencies [145,146,147] in vulnerable subjects. Similarly, other studies described significantly lower levels of KYNA in the peripheral blood of depressed patients with a history of suicidal thoughts [148] and of suicidal patients with schizophrenia [149] compared to the non-suicidal ones. In this framework, two potential mechanisms through which the conversion of TRP to KYN may affect depression and suicidality have been suggested [19]. One theory holds that 5-HT production is decreased as a result of TRP depletion brought on by cytokine activation of IDO [150]. This proposed route is plausible given that hypofunction of the 5-HT system has been linked to recurrent depression and suicide attempts separately [151]. The alternative theory speculates that an increase in KYN metabolites may have a more direct impact on the brain [152]. It is interesting to note that KYNA can also affect the tone of the glutamatergic [153], dopaminergic [154,155], and cholinergic [156] systems, due to its actions as an inhibitor of the α7 nicotinic acetylcholine receptor [157], as well as alter the ability of contextual learning [158], plausibly contributing to executive functioning deficits linked to suicidality [159,160] (see Figure 3). Moreover, KYN metabolites are known to exhibit a wide range of bioactive properties such as oxidant, antioxidant, anti-inflammatory, neurotoxin, neuroprotectant, and/or immunomodulating activity. Their actions depend on the concentration and the cellular environment, and their metabolic system functions under complex positive and negative feedback loops [161]. However, to date, the evidence on the action of KYN metabolites are conflicting, and there is no reached consensus on them.

**Figure 4:**
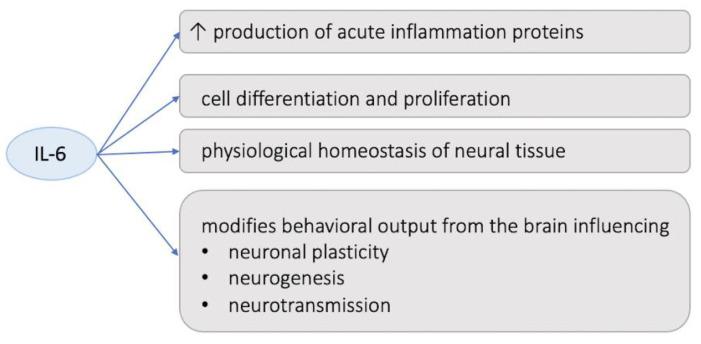
Summarizing figure on the altered IL-6 level’s contribution to suicidality.

Moreover, our results highlighted a positive correlation between the proinflammatory cytokine IL-6 and MOODS-SR total and domain scores, as well as a significant positive correlation between IL-6 and suicidality. The correlation between IL-6 and the MOOD-SR scores is in line with the growing evidence of alterations in IL-6 levels in patients with mood disorders, especially MDD. In particular, according to three meta-analyses, subjects with MDD have higher serum/plasma IL-6 levels than those without depression [162,163,164]. Many items of research have also focused on typifying the depression, reporting significantly higher levels of IL-6 in melancholic depression [165,166,167] and atypical depression [168,169], both characterized by a severe alteration in the circadian rhythmicity pattern, thus validating our results and highlighting a positive correlation between IL-6 and rhythmicity scores. The correlation between IL-6 and suicidality is in line with the branch of scientific literature reporting higher levels of proinflammatory cytokines in the blood, the CSF, and the brain of subjects with a different kind of SB [13,26,27,28,29,30,31,32,33], leading to the suggestion that cytokine stimulation may be involved in the development of suicidal thoughts and behaviors among vulnerable subjects. As a matter of fact, many studies have described higher quantities of IL-6 in the serum and CSF of patients exhibiting suicidal tendencies [47] as well as in children with suicidal tendencies prior to pharmaceutical treatment [49]. Increased levels of IL-6 have been linked to both current and past suicidal tendencies [48], confirming our results (See Figure 4).

**Figure 5:**
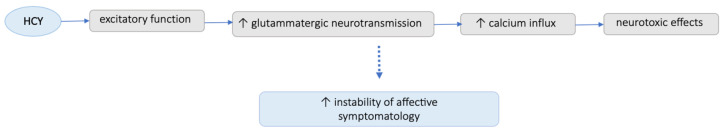
Summarizing figure on the altered HCY level’s contribution to suicidality.

Lastly, our analysis reported a positive correlation between HCY and MOODS-SR depressive component as well as with suicidality scores. Our results are in line with previous research, which reported a substantial correlation between HCY and depression [170,171,172,173] but also identified HCY plasma levels as risk factors for the development of depression [174,175]. This evidence has been recently supported by a meta-analysis that included 46 observational studies [176]. In addition, HCY has been recently linked to suicide risks among patients with depression [177]; this result is in line with our data, which suggested a possible link between HCY levels and suicidality also among subjects in the autism spectrum. One of the possible mechanisms through which HCY levels may be involved in mood balance was hypothesized to lie in the excitatory function of HCY, which leads to an increase in the glutammatergic neurotransmission and, as a result, a calcium influx that has neurotoxic effects, ultimately leading to a greater instability of the affective symptomatology [178,179]. Moreover, a few community-based investigations highlighted a correlation between HCY concentrations and the severity and course of depression symptoms [180,181,182,183], also linking it with an increase in hostility and aggression and even by psychotic symptoms [184,185,186,187]. All this evidence seem to support the hypothesis arising from our results of the existence of a correlation between plasma levels of HCY and suicidality (See Figure 5).

In this framework, research on microbiota has recently gained increasing importance in the field of psychiatric disorder etiopathogenesis. In fact, a rising body of evidence suggests that gut flora and depression are intimately related, and that gut microorganisms may interact with the brain via peripheral inflammation [188,189,190,191,192]. Moreover, intestinal and mucosal layer thinning increases microbiota transmission to the brain and may result in chronic, low-grade inflammation, which is seen in many psychiatric disorders [193]. Furthermore, microbiome composition was examined in the context of reported sleep issues because chronic fatigue has been linked to dysbiosis [194] and insomnia is considered a risk factor for suicidal behaviors [195,196]. Additionally, it has been proven that BDNF in the amygdala and hippocampus, as well as other peripheral and central nervous system events, such as cytokine production, short chain fatty acid release, and microglial maturation and activation, are all influenced by microbe–brain interactions [197,198].

Interestingly, the link between the gut microbiota and inflammation and immunological response has been the subject of extensive research, and numerous studies have demonstrated that the balance between pro- and anti-inflammatory responses in the gut is directly influenced by the gut flora [199]. The so formulated leaky-gut hypothesis describes how the connection between the gut microbiota, CNS, and the periphery leads to a vicious loop that activates inflammatory responses [200], and one of the proposed pathways for the induction of depressive symptoms by pro-inflammatory cytokines include the activation of the enzyme indoleamine 2,3-dioxygenase (IDO), which promotes the metabolic conversion of TRP into KYN [201]. On the other hand, altered KYN/TRP ratio and plasma levels of KYN have been positively linked to the severity of depressive symptoms, the latter also being correlated with suicidality [202], thus suggesting a possible role of microbiota as a marker of suicidal behaviors.

In this framework, our findings suggest, in line with previous studies, that we reconsider the importance of inflammation in the pathogenesis of mental illnesses, in particular the involvement of HCY, the TRP metabolism, and the interleukins system. On the other hand, further research should broadly investigate those alteration in all its pathways and branches. Further research investigating biochemical correlates should move forward acknowledging the potential existence of intertwined relationships between various systems and metabolic pathways, as well as between central and peripheral systems, in shaping suicidal behaviors in all their spectrum of manifestations. Our results also suggest the utility of metabolomics/proteomics approaches in this field of research, which could take into account multiple variables and metabolic signatures. Expanding the research in this area while learning more about the physiological correlates of suicide behaviors may increase the ability to identify useful risk factors and ultimately reveal potential novel therapy targets.

## 5. Limits and Further Directions

Some limitations should be considered in this study. Firstly, the results’ applicability is limited by the small sample size. Additionally, the cross-sectional design of the study did not allow making inferences about possible temporal or causal relationships between the variables. Moreover, there was no information on the dietary status of the subjects, which may have a big impact on TRP levels and metabolism. In addition, the instruments used in the study were self-reported, and as a consequence subjects may have over- or underestimated their symptoms. Finally, this study lacks an investigation of the microbiota activity, which, considering that the bioactivity of the microbiota directly influences serotonin, KYN, and TRP levels, could be of relevance in this context.

Globally, a further in-depth understanding of the pathophysiological mechanisms underlying suicidal thoughts and behaviors is still required, and a special focus should be dedicated to research potential mediators and regulators of the inflammatory response that increase susceptibility or resilience to suicide [34], aiming to develop efficient panels of various biomarkers that could open to a new approach to treatment of suicide with novel treatment targets [187].

Literature regarding the possible therapeutic use of these correlates is still scant. Meanwhile, the possible involvement of glutammatergic routes as well as inflammatory processes in the pathophysiology of psychiatric disorders may eventually promote alternative targets for pharmacotherapy, eventually with a modulatory action on inflammation and glummate pathways [188]. However, further studies are needed in this field.

## 6. Conclusions

In conclusion, although preliminary, our findings confirm the link between suicidality and ASD and provides more evidence regarding the association of suicidality with increased HCY and IL-6 levels as well as with decreased TRP and KYNA ones. These biochemical findings, previously reported by studies among patients with other kinds of disorders, are confirmed herein also among patients in the autism spectrum, further stressing their association with increased suicide risk. Our results also suggest the utility of metabolomics/proteomics approaches in this field of research, which could take into account multiple variables and metabolic signatures. This highlights the need not only to reconsider the importance of inflammation in the pathogenesis of mental illnesses, in particular the involvement of HCY, the TRP metabolism, and the interleukins system, but also to spread the investigation considering these alterations in different metabolic pathways and branches. Future studies should take into account the possible role that metabolic pathways and systems, as well as the interactions between the central nervous system and peripheral systems, may have in influencing suicidal behavior in all of its forms. The ability to identify meaningful risk variables may improve with further studies in this field, also deepening our understanding regarding the pathophysiological correlates of suicide behaviors, which may ultimately offer possible innovative therapeutic targets.

## Figures and Tables

**Table 1:** Comparison of MOODS-SR scores among groups.

MOODS-SR	ASD (n = 24) (Mean ± SD)	BAP (n = 24) (Mean ± SD)	CTL (n = 24) (Mean ± SD)	F	df	*p*
Manic component	22.77 ± 12.20	14.32 ± 9.32	6.12 ± 5.73	18.13	2	<0.001 *
Depressive component	34.32 ± 11.84	16.23 ± 13.73	7.79 ± 5.79	35.39	2	<0.001 *
Rhythmicity	13.00 ± 5.35	9.24 ± 5.36	5.54 ± 4.30	12.74	2	<0.001 *
MOODS total	70.09 ± 23.87	40.62 ± 24.97	19.46 ± 12.68	33.62	2	<0.001 *

* ASD > B AP > CTL; *p* < 0.05 (Bonferroni post-hoc test).

**Table 2:** Comparison of suicidality scores among groups.

Suicidality Sub-Scale	ASD (n = 24) (Mean ± SD)	BAP (n = 24) (Mean ± SD)	CTL (n = 24) (Mean ± SD)	F	df	*p*
Suicidal ideation	1.87 ± 1.73	0.54 ± 1.25	0.12 ± 0.61	12.22	2	<0.001 *
Suicidal behavior	0.50 ± 0.83	0.00 ± 0.00	0.00 ± 0.00	8.62	2	<0.001 *
Suicidality total	2.37 ± 2.24	0.54 ± 1.25	0.12 ± 0.61	14.81	2	<0.001 *

* ASD > BAP, CTL; *p* < 0.05 (Bonferroni post-hoc test).

**Table 3:** Correlations between MOODS-SR scores and biochemical parameters levels.

	Manic Component	Depressive Component	Rhythmicity	MOODS Total
IL-6 pg/mL	0.233	0.428 **	0.538 **	0.422 *
HCY µM	0.119	0.283 *	0.208	0.232
5-HT (PPP) ng/mL	−0.137	−0.188	−0.134	−0.178
5-HT (intra-platelet) ng/mg prot	−0.093	−0.136	−0.064	−0.120
KYN ng/mL	−0.218	−0.099	−0.052	−0.146
KYNA ng/mL	−0.250 *	−0.398 **	−0.315 *	−0.357 **
QUIN ng/mL	−0.069	−0.111	−0.100	−0.104
TRP µM	−0.269 *	−0.259 *	−0.277 *	−0.294 *
BDNF ng/mL	−0.021	0.017	−0.113	−0.022

* *p* < 0.05; ** *p* < 0.01.

**Table 4:** Correlations between suicidality scores and biochemical parameters levels.

	Suicidal Ideation	Suicidal Behavior	Suicidality Total
IL-6 pg/mL	0.476 **	0.289	0.487 **
HCY µM	0.297 *	0.105	0.278 *
5-HT (PPP) ng/mL	−0.084	−0.124	−0.105
5-HT (intra-platelet) ng/mg prot	−0.152	0.044	−0.111
KYN ng/mL	−0.077	−0.215	−0.127
KYNA ng/mL	−0.267 *	−0.116	−0.253 *
QUIN ng/mL	−0.152	−0.159	−0.171
TRP µM	−0.276 *	−0.294 *	−0.132 **
BDNF ng/mL	0.055	0.114	0.079

* *p* < 0.05; ** *p* < 0.01.

**Table 5:** Linear regression analysis with suicidality score as dependent variable and biochemical parameters levels as independent variables.

	b (SE)	Beta	t	*p*	C.I._95%_
Constant	−0.74 (0.55)		−1.36	0.183	−1.85; −0.37
HCY µM	0.18 (0.05)	0.54	3.79	0.001	0.08; 0.28

R2 = 0.290, corrected R2 = 0.270. F = 14.33 (*p* = 0.001). Excluded variables: KYNA, BDNF, IL-6, 5-HT (PPP), 5-HT (intra-platelet), TRP, KYN, QUIN.

## Data Availability

All data generated or analyzed during this study are included in this published article.
